# Inhalable PEGylated Phospholipid Nanocarriers and PEGylated Therapeutics for Respiratory Delivery as Aerosolized Colloidal Dispersions and Dry Powder Inhalers

**DOI:** 10.3390/pharmaceutics6020333

**Published:** 2014-06-20

**Authors:** Priya Muralidharan, Evan Mallory, Monica Malapit, Don Hayes Jr., Heidi M. Mansour

**Affiliations:** 1 Skaggs Pharmaceutical Sciences Center, College of Pharmacy, the University of Arizona, 1703 E. Mabel St, Tucson, AZ 85721-0202, USA; E-Mails: priyam@pharmacy.arizona.edu (P.M.); mallory@pharmacy.arizona.edu (E.M.); malapit@pharmacy.arizona.edu (M.M.); 2 Lung and Heart–Lung Transplant Programs, Departments of Pediatrics and Internal Medicine, the Ohio State University College of Medicine, Columbus, OH 43205, USA; E-Mail: hayes.705@osu.edu; 3 The Davis Heart and Lung Research Institute, the Ohio State University College of Medicine, Columbus, OH 43205, USA; 4 Institute of the Environment, the University of Arizona, Tucson, AZ 85721, USA; 5 National Cancer Institute Comprehensive Cancer Center, the University of Arizona, Tucson, AZ 85721, USA; 6 The BIO5 Research Institute, the University of Arizona, Tucson, AZ 85721, USA

**Keywords:** PEGylated liposomes, PEGylated micelles, sterically stabilized nanoparticles, nasal, lung, aerosol, solid-state PEGylated bilayer nanocarriers, PEGylated inhalation powders

## Abstract

Nanomedicine is making groundbreaking achievements in drug delivery. The versatility of nanoparticles has given rise to its use in respiratory delivery that includes inhalation aerosol delivery by the nasal route and the pulmonary route. Due to the unique features of the respiratory route, research in exploring the respiratory route for delivery of poorly absorbed and systemically unstable drugs has been increasing. The respiratory route has been successfully used for the delivery of macromolecules like proteins, peptides, and vaccines, and continues to be examined for use with small molecules, DNA, siRNA, and gene therapy. Phospholipid nanocarriers are an attractive drug delivery system for inhalation aerosol delivery in particular. Protecting these phospholipid nanocarriers from pulmonary immune system attack by surface modification by polyethylene glycol (PEG)ylation, enhancing mucopenetration by PEGylation, and sustaining drug release for controlled drug delivery are some of the advantages of PEGylated liposomal and proliposomal inhalation aerosol delivery. This review discusses the advantages of using PEGylated phospholipid nanocarriers and PEGylated therapeutics for respiratory delivery through the nasal and pulmonary routes as inhalation aerosols.

## 1. Introduction

The majority of therapeutic agents are hydrophobic or poorly soluble in water, which is a hurdle in drug formulation as it renders the drug less bioavailable. The drugs that make it to the blood encounter further barriers such as chemical or enzymatic degradation, first pass metabolism, inactivation by immune response or clearance by kidney. Macromolecules like protein, peptide, DNA suffer from degradation or inactivation in the body. The most successful systemically administered drugs often end up in liver or spleen due to mononuclear phagocyte system (MPS) uptake. The combination of large molecular size, hydrophilicity, susceptibility to chemical and/or enzymatic degradation, virtually exclude systemic absorption of drugs from most of the mucosal surfaces. All these limitations from the property of therapeutics, in addition to the limitations in the route of administration, propelled to seek suitable alternative means. Inhalation therapies have existed for at least 5000 years [1]. Drug delivery via the lungs is a noninvasive alternative for systemic administration of therapeutic compounds that are poorly absorbed via other mucosal routes.

Drug delivery through the pulmonary route offers several advantages, including increased local concentration of drug, improved pulmonary receptor occupancy, increased absorption due to vast surface area, reduced dose, local and systemic delivery of drug and decreased systemic adverse effect [2,3]. However, drug delivery through the pulmonary route continues to pose challenges like mucociliary clearance and phagocytosis by alveolar macrophage, which can cause drug degradation at the site of absorption [3,4]. Large molecules dissolve in the bronchoalveolar lavage fluid (BALF) and diffuse across the alveolar epithelium; alveolar macrophage presence here can cause drug degradation that will result in reduced bioavailability. Alternately, small molecules are rapidly absorbed through lung epithelium that can be advantageous for immediate release but might not be useful for sustained release. Both cases will end up in increasing dosing frequency which might lead to non-compliance to treatment [5].

This article briefly discusses the development in drug delivery methods that have overcome the limitations of drug administration, with special emphasis on nasal and pulmonary routes.

## 2. Polyethylene Glycol (PEG)ylation Advantages

Polyethylene glycol (PEG) is a neutral, biocompatible polymer with low toxicity and solubility in organic and aqueous solutions, which makes it a popular choice to shield hydrophilic molecule and solubilize hydrophobic molecule. PEG covalently binds to the drug or biological molecule to make it sterically stable. The sterical stability is induced to the molecule by formation of hydration cloud or hydration shell that decreases the zeta potential and prevents interaction of the molecule with any blood components or body cells [6]. PEG can be chemically modified to activate it by groups like carbonate, ester, aldehyde or tresylate which improves the binding of PEG with the drug moiety. Selection of PEGylation site is critical for the protein or polypeptide molecule since different amino acids might have a different outcome upon PEGylation [7,8]. PEGylation chemistry and reaction conditions play a vital role in therapeutic properties of peptides [9,10].

Another remarkable property of PEG is its effectiveness in escaping activation of the complement system, which is the scavenging of some biologically active molecules found in blood through opsonization. Surface charge and hydrophilicity of a molecule plays an important role in opsonization. It has been found in a study involving lipid molecule that PEGylation presented a negative zeta potential to nanoparticles that resulted in weak activation of complement system. The nanoparticles were made of oily triglycerides in the core surrounded by a shell composed of lecithin inside and PEG on the outer surface. The steric hindrance created by PEG molecule was larger than the van der Waals force between the lipid particle and protein, hence protecting the particle from opsonin attack. They also observed that a small-sized curved surface is less favorable for complement activation, which offers spherical nanoparticles a better chance to evade opsonization [11]. These properties of PEG are collectively referred to as the “stealth effect”, while some researchers refer to it as “sterically stabilized molecule”.

**Figure 1:**
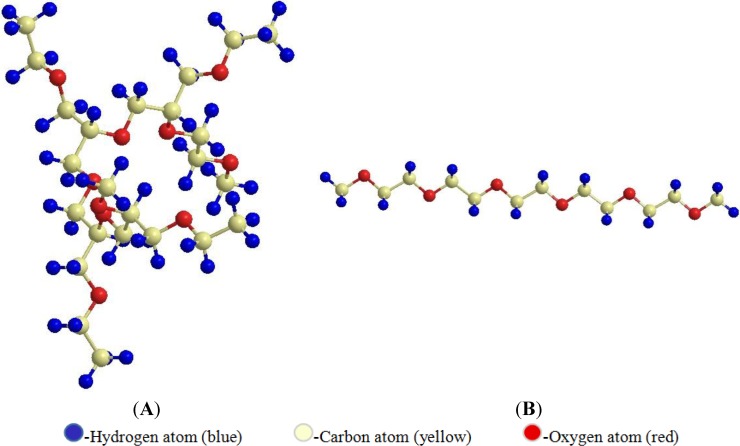
Branched (**A**) and linear (**B**) polyethylene glycol (PEG) (ChemBio 3D Ultra version 13.0.2.3021^©^ 1986–2013 Cambridge Soft, Cambridge, MA, USA).

The steric effect of PEG depends on the size of the PEG chain length. Longer chains provide stronger steric stabilization, as when blood levels of liposome increase when molecular weight of PEG is increased from 750–5000 Da [12]. Molar mass of PEG decides its use and fate in the human body. PEG of size 400–50,000 Da are used for various purposes in the pharmaceutical and medical fields. Linear PEG molecule has a molar mass ≤12 kDa, while branched PEG molecule has larger molar mass ≥60 kDa [9] (Figure 1). The higher molar mass and hydrodynamic volume of branched PEG give more steric hindrance and in turn better shielding characteristics from the immune system [9,13]. It was shown by Youn *et al.* that intratracheal administration of salmon calcitonin showed increased resistance to the pulmonary protein upon increasing the PEG size [14]. Molar mass plays an important role in keeping the PEGylated drug from renal clearance. In a solution, every ethylene glycol in PEG molecule is associated with two or three water molecules which make it bigger than the non-PEGylated drug [9]. Hence, PEG of higher molar mass (20,000–50,000) makes the therapeutic molecule simply bigger than the size the kidney can filter. This property increases the circulation of PEGylated molecule in the blood for a long time, which gives it the opportunity to be used in sustained release pharmaceuticals [6]. Table 1 lists the mechanism by which PEG of different molar mass contributes to sustained release property.

**Table 1:** Molecular mass of PEG and its action *in vivo*.

Molecular Mass (Da)	Conjugated moiety/Delivery	Mechanism [6]
20,000–50,000	Gene delivery, e.g., Oligonucleotides, siRNA	Larger size avoid renal clearance, increased circulation
1,000–5,000	Larger drugs, e.g., Antibodies, nanoparticulate system	Cationic charges are hidden, degradation by enzymes and elimination by RES avoided
3,000–4,000	Oral laxatives	Bulking agent and water retention

RES, reticulo endothelial system.

Absorption characteristics of PEGylated drug conjugates are influenced by the size of the PEG molecule. Gursahani *et al.* demonstrated pulmonary absorption of PEGylated molecules. An increase in molecular weight of PEG leads to increased half-life which can be explained due to increased retention in the lung [15]. The *t*_90_ values of PEG elimination was 2.4 h for 0.55 kDa, 39.9 h for 5 kDa and 41.76 h for PEG 20 kDa, thus suggesting that larger PEG molecules (5 kDa, 20 kDa) can stay in the lungs longer [15].

However, it should be noted that excretion of polymer is not dependent on molar mass of the polymer, but on the hydrodynamic volume of the polymer [6].

Another interesting behavior of the PEGylated molecule is passive targeting of specific cell type or tissue type for drug delivery, especially for diseases like cancer, infection or inflammation. Cancerous cells have poorly developed vasculature that is leaky in nature with a gap of 100 nm to 2 μm; this will allow the drug to leak out of the blood vessel [16]. Additionally, cancer cells have impaired lymphatic clearance that will retain the drug for a relatively longer time [17,18]. This effect is commonly referred to as the “enhanced permeability and retention”, *i.e.*, EPR effect. This increases exposure of tumor cells to drug action.

## 3. PEGylated Drug Delivery

Based on its biocompatibility and amphiphilicity, PEGylation has been applied in biomedical research to suppress graft rejection and immunogenicity and to prolong circulation time in the blood [19]. Pegfilgrastim has increased half-life and reduced dosing frequency than its conventional counterpart [20]. PEG is used extensively in protein and peptide drug delivery in preventing its degradation by proteolytic enzymes, improving solubility, better physical and chemical stability, decreasing renal clearance and imparting lower immunogenicity [9,21,22]. PEGylated drugs are stable over a range of pH and temperature which increases the retention of the drug imparting longer circulation times and makes it a suitable candidate for sustained release drug delivery [23]. When PEG attaches to a drug through covalent bonds, it acts as drug-conjugate, whereas when it attaches via hydrophobic interaction, it acts as drug-carrier system. The majority of proteins are attached to PEG through a covalent interaction [9] that improves the pharmacokinetic profile of protein.

PEGylation has some drawbacks and limitations. Intravenous administration of PEG has caused blood clots in the past. It also caused hypersensitivity reactions when diagnostic agents [24] were intravenously injected [6]. In some cases, PEGylated drug conjugates caused antibody production in the body. The antibody production was noted both in intravenous and subcutaneous administration [6]. Oral delivery of 3.35 kDa PEG has also shown some hypersensitivity reaction, when used as laxative for colonoscopy [6]. An interesting observation was made when salmon calcitonin was made into a PEGylated liposome: the encapsulation efficiency and bioavailability were reduced [25]. Another immunological response of PEG administration was contact dermatitis caused on cutaneous application of PEG of various sizes including 200–400 and 4–20 kDa [6]. However, a byproduct of PEG synthesis, dioxane, is believed to cause it. Some byproducts of PEG are classified as carcinogenic or possibly carcinogenic by the International Agency for Research on Cancer (IARC). Another limitation in the use of PEG is its degradation; it is not degraded in the body. The fate of PEG from PEGylated drug delivery system is unknown. Little information is available about the stress effect on PEG during pharmaceutical processing and in the body. It should be noted that the above effects, excluding fate and degradability, were noted in a small population. Recently, it has been reported that the immune system might start producing anti-PEG molecules against PEG and the formation of vacuoles can limit the extensive use of PEG [24]. Vacuolation in animal tissues by PEG is a serious concern in the use of the polymer. Vacuolation of renal tubules has been reported by a few researchers [24]. Vacuolation is highly influenced by the molecular weight of PEG, which affects the tissue distribution and in turn clearance. Although, there has been no severe effect reports on vacuolation, further research on the safety and effect of vacuolation will facilitate the use of PEG. There have been few cases reporting on the side effects of PEGylated interferon α-2a and ribavirin when injected subcutaneously, which includes adult respiratory distress syndrome, pleural effusion and organizing pneumonia [26,27,28]. There are two cases reporting fatal pneumonitis and pulmonary fibrosis after PEGylated liposomal doxorubicin injectable treatment [29,30], although it was not determined if PEGylation was the reason for pulmonary related complications. Pulmonary administration of PEGylated therapeutics might have the potential to minimize the hypersensitivity and other systemic side effects.

## 4. Phospholipid Nanocarriers

Liposomes are phospholipid bilayer self-assemblies with a vesicle size range of 50–1000 nm [16,31]. A liposome can be a small unilamellar vesicle (SUV), large unilamellar vesicle (LUV) or multilamellar vesicle (MLV). An MLV consists of several concentric (*i.e.*, multilamellar) bilayers with a spherical diameter of 500–5000 nm, whereas LUVs have single large unilamellar vesicle larger than 100 nm and SUVs are single small unilamellar vesicle in the size range of 25–100 nm [31,32]. The majority of clinically approved phospholipids are in the size range of 50–300 nm [31]. The liposomal size is very critical as it determines the fate of the liposome after systemic availability. Liposomes can be physically affected by proteins in the blood, which makes it vulnerable to phagocytic cells and end up being taken up by mononuclear phagocyte system (MPS) [12,16,33]. Particularly larger liposomes are prone to MPS uptake while smaller liposomes escapes detection by opsonin [33]. PEGylation of liposomes introduce stealth behavior to liposome and, therefore, it can escape the phagocytosis by MPS [12,34]. This can keep the liposomal formulation in the blood for a long time, allowing it to be used in treating conditions like cancer. A study on rabbit has shown 275 nm as the maximum size for PEGylated liposome to be circulated long term in the blood [33]. The idea of PEGylation of liposome is similar to red blood cells which circulate for 120 days covered by glycocalyx [35].

As discussed before, surface charge on the liposome can invite opsonin. Neutrally charged liposomes tend to aggregate while negatively charged ones are taken by opsonin [31]. PEGylation increases the hydration shell on the liposomal membrane and prevents aggregation of liposomes; its stealth behavior protects it from opsonin attack. PEG of molecular weight 1000–2000 Da and a density of 5–10 mol% of total lipid is identified to be the optimum characteristic for using with therapeutics [31].

Traditionally, PEG was covalently attached to liposome through chemical moieties such as phosphate, amide, ester, [12]. For example, in proteins, pegfilgrastim, a 20-kDa PEG molecule, is covalently attached to the drug filgrastim by a stable secondary amine bond to the *N*-terminal methionine residue of the protein [36].

Liposomes are successfully being used for drug delivery and vaccine delivery. However, their use for nucleic acid and gene therapeutics continue to be explored [31]. The most commonly used phospholipids in pharmaceuticals are sphingomyelin, phosphatidylcholine, phosphatidylethanolamine, phosphatidylserine and phosphatidylglycerol [31].

The PEG on liposome membrane forms a thick layer around the liposome and is called a hydration layer or fixed aqueous layer. The thickness of this layer increases with PEG chain length which eventually increases the systemic circulation of the liposome [12,31]. Nag *et al.* made an interesting observation where a 1,2-distearoyl-*sn*-glycero-3-phosphoethanolamine-PEG (DPSE-PEG) modified with both 2000 and 500 Da PEG maintained a longer circulation time for the liposome than the individual PEG modification alone [12].

A drug, when associated with liposome, is sequestered away from interaction with its normal site of action until its release from the liposome. Thus, pharmacokinetics of a liposome drug is two-stepped: release of drug from the liposome carrier and action of the drug on the target site. Hence, the rate of release of drug from liposome has effect on the pharmacological action of the drug. This makes the selection of suitable liposome critical criteria for successful drug delivery. Drug property affects liposomal capacity: hydrophilic drug (>1.7 log *p*) is readily retained in liposome and slowly released, hydrophobic drug (<5 log *p*) is readily inserted and retained in liposome; intermediate drug (1.7–5 log *p*) partition between hydrophilic and hydrophobic layer of liposome are rapidly released from the liposomes [35]. PEGylated liposome can passively target tumor cells through EPR effect. The decreased pH (~6.5–7.2) [6] around tumor cells can detach PEG from liposome [37] and the liposome containing drug will elicit its action.

## 5. Nasal Drug Delivery

Intranasal administration has been investigated due to its accessibility and noninvasive nature. The nasal route allows for both local delivery to the upper respiratory tract (*i.e.*, nasal region, nasal tissue, and nasal fluids), noninvasive systemic delivery, and noninvasive CNS (central nervous system) delivery of drugs, due to the large surface area and highly vascularized nature of the nasal cavity and direct access to the olfactory region [38]. Drug administration through the nasal route has shown its success throughout the years, allowing for the avoidance of first-pass effect, reducing systemic side effects, bypassing blood–brain barrier (BBB) [39] and increasing bioavailability [38,40]. PEGylation of therapeutics has been shown to increase stability, improve pharmacokinetic parameters, and can enhance nasal absorption [40,41]. Conjugating PEG has been shown to reduce adverse effects such as chitosan cytotoxicity while maintaining membrane permeability [42]. PEGylated molecules delivered through the intranasal route can involve vaccines, protein/peptides, and hormones for systemic and CNS delivery. Table 2 lists examples of PEGylated intranasal therapeutics.

## 6. Nasal Delivery of PEGylated Therapeutics for Upper Respiratory Tract Delivery and Noninvasive Systemic Delivery

PEGylation of glucagon-like protein-1 (GLP-1) for the treatment of type 2 diabetes *in vivo* in diabetic mice has been reported where GLP-1 was conjugated with PEG (molecular weight 1, 2, or 5 kDa) [41]. The PEG-2000 conjugate was shown to have the greatest hypoglycemic score. PEGylation of GLP-1 prolonged the half-life of GLP-1 and decreased renal clearance rate [41]. This study has shown that the half-life was directly related to the PEG chain length; however, the PEG chain length was inversely related to nasal absorption rate and biological activity of the peptide [41]. PEGylation enabled the avoidance of protein degradation from nasal mucosal enzymes. Post-prandial hyperglycemia was effectively stabilized *in vivo* in mice by the intranasal administration of PEGylated GLP-1 proteins [41]. Another study investigated a GLP-1 receptor agonist, exendin-4 (Ex4), in type 2 diabetic mice for its glucoregulatory effects [40] where Ex4 was conjugated with PEG (molecular weight 1, 2, or 5 kDa). PEG-1000 demonstrated the greatest biologic activity of Ex4, as steric hindrance may have interfered with the activity of Ex4 with PEG-2000 and -5000 conjugates [40]. Keeping constant with previous results, higher molecular weights of PEG have decreased nasal absorption, but also exhibit a longer half-life [40]. Another study reported PEGylated protein conjugate for systemic delivery by investigating the absorption potential of polyethylene glycol (PEG)-modified salmon calcitonin (sCT) in rats administered via the nasal route [43]. When compared to unmodified sCT, PEGylated sCT exhibited a prolonged serum calcium-lowering effect. PEG conjugated with salmon calcitonin was used at molecular weights of 2, 5, and 12 kDa. The PEG-2000 conjugate exhibited the greatest hypocalcemic effect, and higher molecular weights of PEG (greater than 5 kDa) may have limited absorption. This study exhibited that it can be absorbed systemically, and the hypocalcemic effect of PEGylated sCT was affected by the length of conjugated PEG [43]. Vaccination through the nasal route for the prevention of certain infectious diseases has been studied extensively leading to a few currently marketed nasal vaccine products [44]. Nasal delivery of vaccine has to be more effective than intramuscular or oral route of delivery in bypassing pre-existing immunity [45].

**Table 2:** Examples of PEGylated nasal drug delivery systems [39,40,41,43,45,46].

Therapeutic	Prevention/Treatment	Absorption Target	Carrier/Conjugate
Viral DNA	Vaccinations	Systemic	PEGylated oligonucleotide
Glucagon-like protein-1	Diabetes mellitus type II	Systemic	PEGylated protein hormone
Calcitonin	Osteoporosis, bone diseases	Systemic	PEGylated protein hormone
Growth factor	Alzheimer’s disease	CNS	PEG-PLGA

Abbreviation: CNS, central nervous system; PLGA, poly(lactic-co-glycolic acid).

## 7. Noninvasive Delivery to the Central Nervous System (CNS) through the Intranasal Route

PEGylated drug delivery through the nasal route has considerable potential in regards to the transport of drugs directly to the brain through the olfactory region [47]. PEG–PLGA (PEG–poly(lactic-co-glycolic acid)) nanoparticles have demonstrated effectiveness in delivering basic fibroblast growth factor (bFGF) directly to the brain for treatment of Alzheimer’s disease. The PEG–PLGA nanoparticle formulation resulted in higher drug concentration in the brain than unmodified nanoparticles and intravenous administration [39]. Previous studies had shown that PEG–PLA (PEG–poly(lactic acid)) nanoparticles covalently bound to wheat germ agglutinin (WGA) resulted in an increase in brain uptake of drugs when administered intranasally. These studies demonstrated negligible nasal cilia toxicity [46,48]. The studies were also directed to explore the possible nasal administration of nanoparticles for drug transport to the brain. These studies have shown success in CNS delivery through not only the olfactory region, but also the transgeminal nerve pathway [46]. Direct transport to olfactory epithelium cells may be developed by PEGylating immunoliposomes. By conjugating monoclonal anti-glial fibrillary acidic protein (GFAP) antibodies to PEG–liposomes, this resulted in the formation of a high-specificity delivery system, which, therefore, directs the transport of diagnostic and medicinal drugs to olfactory ensheathing cells. In this study, PEG-2000 was conjugated to immunoliposomes that specifically bind to olfactory ensheathing cells (OEC). The vectors used were monoclonal antibodies against GFAP. Although this study was performed *in vitro*, this demonstrates the potential of PEGylated liposomes for nasal drug delivery targeting the CNS [47].

## 8. Pulmonary Administration of Nanocarriers and Inhaled Nanocarrier Liquid Aerosols

The airway offers ease of local and systemic drug delivery by virtue of noninvasiveness. Anatomical and physiological characteristics favor absorption through the thin alveolar epithelium (0.1–0.5 μm) which possesses high permeability. Other notable features of pulmonary route include large surface area (~140 m^2^), relatively low enzymatic activity, and direct entry to the systemic circulation through the thin alveolar epithelium, thus avoiding the hepatic “first-pass” metabolism. The respiratory tract is complex with its bifurcation and dynamic clearance mechanism. It is crucial that the drug particles reach the site of action in spite of the barriers. The key factors that impact drug deposition in lungs following aerosolization are aerosol particle properties (*i.e.*, aerodynamic particle size, particle density, particle size distribution, surface morphology, and particle shape), anatomy of the respiratory tract, and the patient inspiratory flow rate [49]. There are five mechanisms of aerosol particle deposition with three major mechanisms (*i.e.*, inertial impaction, sedimentation by gravitational settling, and diffusion by Brownian motion) and two minor mechanisms (*i.e.*, interception and electrostatic deposition) [1,49]. Inhaled aerosol particles with an aerodynamic particle size of greater than 10 μm will deposit in the oropharygeal region by inertial impaction; whereas sedimentation by gravitational settling governs aerosol particle deposition in the mid-lung and small airways for 1–5 μm aerodynamic-sized particles and smaller particles of aerodynamic size <1 μm deposit by diffusion/Brownian motion [1,49].

Mucus is a thick, viscous, elastic liquid [50] secreted by the ciliated cells that lines the outer lung respiratory track. The cells are found from trachea to terminal bronchiole that creates a blockade for drugs to reach its site of action. Drug particles that get entrapped in the mucus layer are removed by the mucociliary clearance where the constantly beating cilia push the entrapped drug to the pharynx [51]. Some important characteristics of mucus include viscosity, elasticity, adhesivity, permeability and clearance rate [51]. Composition and characteristics of mucus vary based on its anatomical location and physiological condition. In the lungs, alveolar type II cells synthesize lung surfactant that is primarily made of phospholipids and protein. It is the first line of defense to inhaled bacteria and other pathogens [51]. Alveolar macrophage in the alveoli of lungs is the body’s immune response that becomes a hurdle to drug delivery. The macrophages scavenge anything that is recognized as a foreign substance in the body [52].

The nanocarrier system (colloidal carrier) comprises nanosized particles and possesses tremendous potential to carry the drug or diagnostic imaging agents. It is of interest to use nanocarriers for cancer treatment primarily due to extravasation property of nanoparticles into tumor cells. In pulmonary drug delivery, nanocarriers can be used due to their EPR effect [53] and the ability to diffuse across the mucus layer as drug carrier. Nanoparticles offer many advantages as a drug delivery system including: (i) increased solubility of hydrophobic drugs; (ii) sustained release kinetics; (iii) uniform distribution; (iv) passive targeting; (v) enhanced permeability; (vi) ability to target cell internalization pathway; (vii) targeted delivery for tumor; and (viii) reduced dosing frequency and improved patient compliance [3,54].

**Figure 2:**
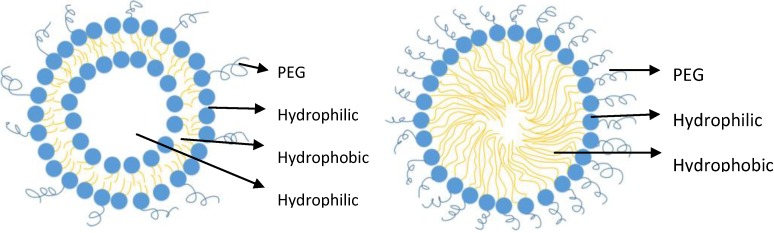
PEGylated Liposome (**left**) and PEGylated Phospholipid Micelle (**right**) (Microsoft Visio professional 2013, Redmond, WA, USA).

Briefly, there are different types of nanoparticulate drug delivery systems (nanocarrier) which include polymeric nanoparticles (e.g., nanosphere, nanocapsules, dendrimers, polymeric micelles), lipid nanoparticles (e.g., solid lipid nanoparticles, liposomes, fatty acid micelles), lipopolymeric nanoparticles (e.g., PEGylated phospholipid micelles, PEGylated liposomes), carbon nanotubes, and nanocrystals [16,55,56]. For pulmonary delivery, polymeric nanoparticles are made up of biodegradable or biocompatible polymer materials [3]. Polymeric micelles are made of amphiphilic molecules that form a core and shell structure with a hydrophobic core and a hydrophilic surface. A PEGylated liposome is made of a concentric phospholipid bilayer (*i.e.*, lamellar) with a hydrophilic core (Figure 2) and is typically a mixture of phospholipid (major component) and PEGylated phospholipid (minor component) [16]. With only PEGylated phospholipid present, a PEGylated micelle exists (Figure 2). Inorganic nanoparticles are beyond the scope of this article, and hence, will not be presented. For further details on different types of nanoparticles, the reader is referred to textbooks on the topic [57].

The nanoparticle carriers can be formulated into solution or suspension and delivered by a nebulizer or pressured metered dose inhaler (pMDI), while solid state nanoparticles as powders are delivered via a dry powder inhaler (DPI) [32]. Nanoparticle size of 40–200 nm has exhibited cellular uptake *in vitro*. Nanoparticles can envelope the drug from degradation, increase solubility, as well as be used for sustained release delivery and targeted drug delivery [58].

Despite the advantages that the nanoparticle offers, it is susceptible to innate immune response, and mucus layer is a barrier for nano-drug delivery to the lung. As previously discussed, surface modification of the drug molecule or drug carrier can lead to avoidance of innate immune response and increase lung entrapment [59]. Simply put, PEGylation imparts the stealth characteristics to nanoparticles and improves its circulation time in the blood. A wide variety of drugs including corticosteroid, anti-inflammatory, vaccines, and anti-cancer agents have been studied for their efficacy through the pulmonary route. Inhalation is the most promising alternative route for proteins and peptides than injection, as lungs are permeable to macromolecules [60]. However, inhaled peptides create an immune response and are broken down by pulmonary peptidase, particularly serine peptidases and aminopeptidases. Thus, PEGylation of drug or protein offers an ideal means to deliver it to the lungs, increases the half-life and bioavailability of drug in the body [7,34,61].

Increasing PEG chain increases stability of the protein molecule; yet, it also interferes with the biological activity of protein. Hence, it is important to optimize the size of PEG to strike the right balance between biological activities and improved pharmacokinetics [14]. Phospholipid has shown improved particle migration to the lung periphery due to reduction in surface tension provided by the lung surfactant [62]. PEG chain length can potentially offer enhanced mucus penetration by phospholipid spreading and PEG penetration, controlled drug release, and “stealth” property to escape the phagocytosis by immune cells [62]. In another study conducted by Ibricevic, they modified the outer shell of cationic shell cross-linked knedel-like nanoparticles (cSCK-NP) by PEGylation, to identify a way to overcome physiological barriers in alveolar epithelium to treat acute and chronic lung injury. Depending on the extent of PEGylation, cSCK-NP delivery to airway improved by decreasing the inflammatory response. However, the exact mechanism of how PEGylation decreases the inflammatory response is not well understood [63]. The size of a PEGylated drug/NP also influences the cellular penetration, residence time, and deposition in the alveoli which are important aspects of inhalation of drugs for systemic delivery through the pulmonary route.

Liposomal pulmonary drug delivery offers several advantages [32]. The phospholipid, dipalmitoylphosphatidylcholine (DPPC), is the major phospholipid component of lung surfactant and is essential for proper lung surfactant function. In addition to DPPC, another phospholipid used in liposomal formulations for pulmonary delivery is distrearoylphosphatidylcholine (DPSC). Beclomethasone dipropionate, a pulmonary corticosteroid, encapsulated in 1,2-distearoyl-*sn*-glycero-3-phosphoethanolamine (DSPE)-PEG as sterically stabilized micelles for pulmonary delivery [64]. When low molecular weight heparin was formulated with a PEGylated liposomal carrier, its half-life and *in vivo* lung absorption increased with prolonged blood circulation [65]. The circulation time *in vivo* was influenced by PEG size where PEG 2000 Da had a better circulation time over PEG 5000 Da [65].

Polymeric micelles are colloidal nanocarriers made of amphiphilic polymers where the hydrophobic part comprises the dense core while the hydrophilic part forms the outer shell. This will increase the solubility of poorly water-soluble therapeutics such as gene delivery (siRNA), macromolecules (protein, peptide, heparin) [4,16]. The small size of the micelles permits their extravasation and accumulation in a variety of pathological sites such as tumors [64]. Selection of polymer is important, structural compatibility between the hydrophobic core and the drug molecule determines the encapsulation efficiency of the micelle [66]. Despite the many natural and synthetic polymers available, poly(ethylene glycol) is a common drug delivery polymer because it is non-ionic, biocompatible, and has low fouling, low toxicity, in addition to a stealth behavior which imparts low immunogenicity and antigenicity. Due to its steric hindrance, PEG can prevent aggregation of micelles in solution, thereby enhancing physical stability. The typical size of the polymeric micelle ranges between 10 and 100 nm [4]. A comparative study was conducted by Sahib *et al.* where they compared budesonide suspension (Pulmicort Respules^®^) in the market with sterically stabilized phospholipid nanomicelle of budesonide (BUD-SSNM) made of DSPE-PEG. The comparison brought out interesting results; the particle size of Pulmicort Respules^®^ was 2–3 μm, whereas SSNM was 20.45 ± 1.64 nm, which suggest that the suspension generates larger particles while aerosolized SSNM can make smaller and more abundant particles [4]. It was also noted that the after six hours, 90% of budesonide was released from Pulmicort Respules^®^, while less than 35% of budesonide was released from BUD-SSNMs, with a complete release within six days. Another observation was made regarding the pharmacodynamic behavior where BUD-SSNM showed a significantly longer duration of action in inhibiting inflammatory cell infiltration in the airways that were prolonged for up to 24 h, compared with Pulmicort Respules^®^ [4]. Hence, SSNM has a better-sustained release characteristic than suspension.

PEGylated phospholipid forms micelles similar to polymers, where the lipid contributes to the hydrophobic part of the micelle [4,67]. Sterically stabilized micellar (DSPE-PEG 2000) nanocarriers encapsulating GLP-1 in saline, GLP-1 underwent secondary conformation which increased the α-helicity of the peptide, which improves the activity of the peptide [68]. Paclitaxel loaded into DSPE-PEG 5000 micelles was formulated for pulmonary delivery as micelles with an average size of 5.0 ± 0.7 nm [69]. Owing to the small size, these micelles escaped alveolar macrophage phagocytosis. When the same formulation was administered intravenously, most of the drug ended up in the liver or spleen. The study showed that PEG–phospholipid micelles helped to attain high blood levels of paclitaxel when administered locally to lung, which is needed for treating lung cancer [69]. It can be inferred from the two studies that hydrophilic PEG associated with water, while hydrophobic DSPE with the drug formed the core of micelles. The main mechanism of drug release from micelle core would be through diffusion.

A well-defined structure, monodispersity of size, surface functionalization capability and stability makes polymeric dendrimer a suitable candidate for drug delivery [5,16]. Dendrimers are highly branched macromolecules; however, the size and shape of dendrimers can be precisely controlled. In a study containing polylysine dendrimer solution, pharmacokinetics of PEGylated dendrimers were characterized after pulmonary delivery in rats. The results suggest that PEGylated dendrimers have the potential to control drug release and absorption kinetics following pulmonary delivery. A correlation between absorption property and PEGylated dendrimer molecular weight was observed, which suggests that smaller dendrimers (11 and 22 kDa) had relatively better absorption but limited lung retention when compared to larger dendrimers (78 kDa) which was not absorbed into the blood although it lasted in the lung for 168 h [5]. This helps in choosing the appropriate dendrimer size for the relevant purpose: small dendrimers can be used for rapid onset of systemic action, while larger dendrimers can be used for prolonged release. Bai *et al.* did the first study to report negatively charged low molecular weight heparin (LMWH), with a molecular weight of 5 kDa, can be encapsulated in mPEG-conjugated dendrimers [70]. Upon PEGylation, the particle size of dendrimers increased by four-fold compared their non-PEGylated formulation. The increase in size increased the inner cavity space, which can accommodate larger molecules, and subsequently increase drug-loading capacity. The increase in entrapment efficiency of LMWH was because of electrostatic interactions and/or hydrogen bonding of the drug with dendrimeric core and PEG arms, respectively. The entrapment of drug in the dendrimeric core resulted in increased half-life and absorption of the drug through the pulmonary route. Hydrophobic agents such as adriamycin, artemether, methotrextate, rifampicin and pyrene are reported to interact with the hydrophobic dendritic core [70]. Table 3 lists examples of such systems that were successfully formulated and tested in a rodent model.

Pulmonary drug delivery has demonstrated promising results in chemotherapeutic delivery. Successful delivery of doxorubicin via an intracorporeal nebulizing catheter in rats was demonstrated to be successful using transferrin-conjugated PEGylated liposomes [71]. HIV-1 TAT, trans-activator protein was conjugated with polyethyleneimine (PEI) through PEG (3.4 kDa) forming TAT-PEG-PEI and showed enhanced transfection efficiency by intratracheal instillation [72]. The addition of the PEG was shown to increase stability of DNA in the pulmonary environment as well as improve transfection efficiency. The TAT-PEG-PEI construct offers promise for the delivery of DNA to treat local diseases in the lungs.

**Table 3:** Examples of PEGylated therapeutics and PEGylated nanocarriers studied in rodent models.

Drug	Conjugation	Carrier	Admin Route	Disease	PEG Molecular Mass (Da)
Budesonide	DSPE-PEG	PEGylated micelle	Nebulizer	Asthma COPD	5,000 [4]
Lysine	Lys_16_ PEG	PEGylated Dendrimer	Intra tracheal	–	200 and 570 [5]
SiCTGF	PDMAEMA/PDMAEMA-b-PMAPEG	PEGylated siRNA polymeric complex	Intracheal instillation	Pulmonary fibrosis [73]	–
LMWH	DSPC:DSPE-PEG	PEGylated liposome	Intra tracheal	DVT, pulmonary embolism	2,000–5,000 [65]
Paclitaxel	DSPE-PEG	PEGylated micelle	Intra tracheal	Lung cancer	5,000 [69]
LMWH	DOTAP, cholesterol and DSPE-PEG-2,000	Cationic PEGylated liposome	Intra tracheal	DVT	2,000 [74]
Glucagon like peptide-1 (GLP 1)	mPEG	PEGylated protein	Intra tracheal	Type II diabetes	2,000, 5,000, 10,000 [75]
Salmon Calcitonin	mPEG-SPA	PEGylated protein	Intra tracheal	Osteoporosis	1,000, 2,000, 5,000 [14]
Ciprofloxacin	PC, cholesterol, diacetylphosphate, DSPE-PEG	PEGylated liposome	Intra tracheal	Respiratory infection	2,000 [34]
LMWH	PAMAM-PEG	PEGylated Dendrimer	Intra tracheal	DVT	2,000 [70]

Abbreviations: DSPE, 1,2-distearoyl-*sn*-glycero-3-phosphoethanolamine; PEG, poly(ethylene glycol); COPD, chronic obstructive pulmonary disease; SiCTGF, small interfering conncetive tissue growth factor (siRNA); PDMAEMA, poly(dimethylamino) ethylmethacrylate; b-PMAPEG, poly (ethyleneglycol-*a*-methylether-*u*-methacrylate) (b-represents binary complex); LMWH, low molecular weight heparin; DVT, deep vein thrombosis; DOTAP, 1,2-dioleoyl-3-trimethylammoniumpropane (chloride salt); SPA, succinimidyl-activated monomethoxy; PC, phosphatidylchloine; PAMAM, polyamidoamine.

## 9. Dry Powder Inhalers (DPIs)

According to the World Health Organization, tracheal, bronchus, lung cancer, chronic obstructive pulmonary disease (COPD) and lower respiratory infections are listed among the 10 leading causes of death worldwide (15.2%) [76]. Hence, there is much motivation and rationale in improving drug delivery to lungs for local action. Many effective pulmonary drugs are lipophilic molecules, which make them good candidates for liposomal dry powder formulation for pulmonary nanomedicine delivery with controlled-release properties. As a solid state formulation, dry powder inhalers offer advantages like improved stability, opportunity to modify the particles to suit different needs, minimized variation in aerosolization, propellant free, portable, ease of use and low-cost devices [77,78]. Among the available pulmonary delivery devices, pMDI and soft mist inhalers have a limitation on the liquid volume that can be delivered in a single puff, this ranges anywhere from 25–100 μL. The alternate method is using a nebulizer to deliver a large aerosol dose over long treatment times. However, a nebulizer can cause inconvenience to patients by virtue of its large size, requirement of additional power source, noisy compressor and need for maintenance. On the other hand, DPIs have the efficiency to deliver higher doses. For example, the first needle-free inhaled powder delivered 1–3 mg of insulin [77]. Liposomes are a thermodynamically stable system that allows the formulation of dry powder. Solid state liposome, where it loses water, is called liposphere or proliposomes [32].

The rise of antibiotic resistance in recent years has led to a shortage of antibiotics capable of treating bacterial infections [79]. It has been suggested that the development of new and more effective antibiotics may be spurred through research into new delivery systems [80]. Due to its decreased systemic adverse effect, the pulmonary route is preferred for antimicrobial delivery to treat lung disease since this will reduce systemically induced antibiotic resistance. The operating principle behind the dry powder inhaler is that micron or nano-sized particles are blended with an inert carrier particle to give it bulk that can flow on inspiration; this generates the aerosol that is inspired by the patient. During inspiration, the drug particle separates from the carrier, owing to the size of the drug particles that reach the lungs [78]. The carrier particle plays a key role in the drug delivery, hence selecting a suitable carrier is critical for the performance of DPI. The most popular carrier is lactose; other carriers are mannitol, trehalose, erythriol, and sorbitol. An *in vitro* study reported that ciprofloxacin delivery via a dry powder inhaler (DPI) using PEG (8 kDa) has particle characteristics suitable for the treatment of infections found in cystic fibrosis patients [81].

Several drugs have been successfully formulated into inhalable non-PEGylated proliposomal dry powders [32], which include but are not limited to budesonide, beclomethasone and formoterol, salbutamol sulfate, ketotifen fumarate, dapsone, amiloride hydrochloride, tacrolimus, and cyclosporine A. Curcumin has been shown to have anti-inflammatory and antioxidant properties that may be promising in the treatment of cystic fibrosis, asthma, COPD, and other pulmonary diseases [82,83,84]. Curcumin was developed as a DPI using m-PEG (5 kDa) and both particle characterization and *in vitro* studies showed promise for its sustained pulmonary drug delivery [85]. Meenach *et al.* successfully produced and optimized DPPC:DPPE (dipalmitoylphosphatidylethanolamine)–PEG as PEGylated phospholipid bilayer nanocarriers in the solid-state with varying PEG-chain lengths (2, 3 and 5 kDa) with essential characteristics necessary for inhalation as DPIs [86]. These PEGylated phospholipid bilayer nanocarrier DPIs consisted of DPPE-PEG 5 mol% mixed with phospholipid DPPC (dipalmitoylphosphatidylcholine) 95 mol%. In addition, Meenach, *et al.* also encapsulated paclitaxel, a first-line lung cancer chemotherapeutic drug, at various molar ratios with DPPC:DPPE–PEG as PEGylated phospholipid bilayer nanocarriers in the solid-state with varying PEG-chain lengths as DPIs for use in targeted lung cancer treatment [62].

PEGylation technology of proteins (e.g., insulin and human growth hormone) for inhalation as dry powders has been reported in order to achieve both efficient absorption across lung epithelia and extended serum concentrations [87]. Table 4 lists some PEGylated aerosol nanoparticles for pulmonary inhalation delivery.

**Table 4:** Examples of aerosolized PEGylated nanocarrier delivery to the lungs.

Aerosol Type	PEG Length	Therapeutic	Carrier	Disease	Reference
DPI	2, 3, and 5 kDa	Paclitaxel	PEG	Lung cancer	[62]
DPI	5 kDa	Curcumin	PLGA–PEG chitosan	Asthma, COPD, Cystic Fibrosis	[85]
DPI	8 kDa	Ciprofloxacin	PEG	Respiratory infections	[81]
Colloidal Dispersion	2 kDa	Ciprofloxacin	PEGylated liposome	Respiratory infections	[34]
DPI	–	Insulin	PEG	Diabetes mellitus	[87]

Abbreviations: COPD, chronic obstructive pulmonary disease; DPI, dry powder inhaler; PLGA, poly(lactic-co-glycolic acid).

## 10. Conclusions and Future Directions

PEGylation has some known limitations such as proper PEGylation site, preparation chemistry, and side effects in relation to the route of administration. The benefits of PEG outweigh the limitations for the continued use of PEG in pharmaceuticals and diagnostic agents. Several studies have explored the potential of drug delivery through the nasal route, but few have reported on how PEG may be utilized in this manner by either conjugating onto a liposome or directly onto a protein. The noninvasive nature of nasal delivery can provide unique opportunities for the design and development of more intranasal vaccinations and PEGylated therapeutic peptide nasal formulations. Future directions in nasal delivery of PEGylated drugs include expanding research for pain, migraines, other CNS disorders, protein and peptide delivery, and vaccinations. PEGylated drugs delivered through lungs offer several unique advantages. Research on PEGylated nanocarriers as dry powder inhalers is a very promising yet still emerging field of study. Further research in the exciting area of inhaled PEGylated nanocarriers as DPIs offers unique opportunities in pulmonary inhalation aerosol delivery.
